# Immune Regulation during Helminth Infections

**DOI:** 10.1371/journal.ppat.1003250

**Published:** 2013-04-18

**Authors:** Natasha M. Girgis, Uma Mahesh Gundra, P'ng Loke

**Affiliations:** Department of Microbiology, New York University, New York, New York, United States of America; University of Wisconsin Medical School, United States of America

## The Co-Evolution of Helminths and the Mammalian Immune System

Helminth is a nonphylogenetic term that refers to multicellular animals (or metazoans) that have adopted a parasitic lifestyle in mammalian hosts. They are more commonly referred to as parasitic worms. These worms generally (with the exception of *Strongyloides stercoralis*) cannot replicate within the host, and they have evolved distinct methods to co-exist with their host through the activation of an immune regulatory network. Until quite recently in human history, the majority of *Homo sapiens* were likely colonized by one type of worm or another, just like many mammals are in the wild. This close relationship has led to the evolution of “disease tolerance” by the host [1], [2], in the presence of these parasites [3]. In other words, the mammalian host has evolved mechanisms to minimize the virulence of helminths, even without necessarily reducing worm burden. While helminths cause disease in hundreds of millions of people [4], nonetheless a large proportion of infected individuals are relatively tolerant to colonization with helminths.

## Heterogeneity in Susceptibility to Infection and Pathology

Maintaining variation in immunity to any parasite is critical toward the survival of a host population. In an endemic population, a large proportion of helminth-infected individuals are relatively asymptomatic. Pathology often occurs in individuals that have a reduced immunity and are therefore highly susceptible to infection with very high worm burdens. Seventy percent of the worm burden in a population may occur in only 15% of the infected individuals [4]. Pathology can also occur in individuals that are very immunologically reactive, despite having low worm burdens [5], [6]. In this case, a breakdown of the immune-regulatory environment established by the host and the parasite may have occurred. Maintaining a disease-tolerant or asymptomatic state requires an appropriate immune regulatory relationship between each host and helminth, which is unlikely to occur for every individual host. Hence, there is a strong need to understand the natural variation in immune responses against helminths [7]. There is evidence that resistance to helminth infection could improve survivability during harsh conditions, but brings with it the cost of autoimmune susceptibility and reduced reproduction [8]. As clinical trials reintroducing helminths as treatment for autoimmunity are ongoing [9], [10], there is an urgency to understand heterogeneity in immune responses against helminths in order to personalize treatment regiments to maximize clinical benefit.

## Type 2 Responses Minimize the Virulence of Helminth Infections

A key component of the immune system that has evolved to minimize the virulence of helminths is the type 2 (or T_H_2) response [11]. Instead of giving us allergies and asthma, the type 2 response likely evolved both to provide resistance by limiting the number helminths that can live in our intestinal tract [12] and to repair the tissue damage that is caused by the helminths that have colonized our tissues [3]. This response is characterized by the production of cytokines such as interleukin-4 (IL-4), IL-5, IL-9, and IL-13. While it is still unclear how this response is initiated during helminth infection, a broad range of effector mechanisms are activated by these cytokines [11], [13].

Signaling through IL-4Ra and STAT6 in intestinal epithelial cells (IECs) promotes goblet cell differentiation and increases mucus production, as well as increases proliferation and turnover of the IECs [12]. This may help maintain the mucosal barrier and limit aberrant responses triggered by the gut bacteria [10]. Increased contraction of intestinal muscles and the activation and release of mast cell proteases that can increase fluid flow into the lumen may also help flush the worms out of the gut [12]. Macrophages are alternatively activated by IL-4Ra/STAT6 signaling to adopt an anti-inflammatory tissue repair function [3]. The type 2 response will also increase secretion of Immunoglobulin E (IgE) by B cells that can in turn activate cells that express Fc receptors (FcRs), such as basophils, eosinophils, and mast cells to amplify the type 2 response by producing more IL-4. The absence of this type 2 response during helminth infections in mice is often associated with lethal sepsis from compromised gut integrity and leakage of gut bacteria [3].

## Host Mechanisms of Immune Regulation during Helminth Infection

During chronic helminth infection, peripheral T cells from infected patients are unresponsive to stimulation with parasite antigens, and responses to other antigens are also reduced [6]. This observation led to studies to define the immune regulatory mechanisms at play during helminth infection. In addition to the T_H_2 response described above, regulatory T cells [14], regulatory B cells [15], and alternatively activated (or M2) macrophages [16] were identified as key components of the immune regulatory network functioning during helminth infections [13].

Foxp3+ Treg cells expand during helminth infections and may promote the survival of helminths as well as limit immune-driven pathology [14]. Depletion of these Treg cells can increase resistance to the parasites and reduce worm burden, but can also lead to increased immune-driven pathology [14]. IL-10 producing regulatory B cells was first identified to play an important role in limiting disease severity during autoimmune diseases and then later found to be induced by helminths [15]. In several mouse studies whereby helminths could suppress allergic inflammation, suppression could be reversed by depleting these B cells or transferred to a recipient animal by transferring B cells [15]. Alternatively activated macrophages that are expanded during helminth infections have been shown to promote Foxp3+ Treg differentiation [17] and, by up-regulating arginase 1, play an important immune regulation and tissue repair role, respectively, by competing for L-arginine and generating proline [13], [16]. The expansion and increased functionality of these regulatory cells during helminth infection may be responsible for the bystander suppression of autoimmune diseases, which has been noted in several study cohorts [5], [18]. Additionally, activation of this immune regulatory network likely contributes toward the deficiency in generating protective immunity when exposed to natural helminth infection [6].

## Helminth Products That Enhance Immune Regulation

In order to promote the host immune regulatory network described above, helminths have evolved the production of immuno-modulating molecules that are just being elucidated [19], [20]. A few examples include a TGF-β-like ligand identified from the secreted products of the intestinal nematode parasite *Heligmosomoides polygyrus bakeri* can induce the differentiation of regulatory T cells [20]. A glycoprotein secreted *by Schistosoma mansoni* eggs called omega-1 has been shown to condition dendritic cells to promote T_H_2 differentiation [19]. The phosphorylcholines covalently attached to the N-type glycans of ES-62, a protein secreted by filarial nematodes, have been shown to suppress inflammatory responses [19]. While the mechanisms of action are still unclear, some of these molecules have been shown to act through Toll-like receptors (TLRs) and can in turn suppress pro-inflammatory responses triggered by other TLR ligands such as lipopolysaccharide (LPS) [19]. Since many helminths share the intestinal tract with commensal bacteria and can cause tissue damage to the intestines, there could be an evolutionary advantage toward suppressing the host responses against bacterial TLR ligands and preventing sepsis from a leaky gut.

## Suppression of Autoimmunity by Helminth Infection

In the last 50 years there has been an exponential increase in various diseases of immune dysregulation in the developed world. Regions of the world where helminth parasites are still endemic because of poor sanitation have a lower prevalence of allergies and autoimmune diseases [5], [21]. Studies on individuals from endemic regions treated with anti-helminthic medications have generally shown an increase in allergen reactivity posttreatment [5]. Through mouse models, various helminths have been shown in many studies to suppress the symptoms of many different types of experimental autoimmune diseases (e.g., experimental autoimmune encephalomyelitis, type 1 diabetes, arthritis, and colitis) as well as allergic conditions of the skin, intestines, and the airways [21]. Based on these studies, investigators began to conduct clinical trials introducing live helminths to patients with autoimmune diseases [9], [10], [21]. *Trichuris suis* (the pig whipworm) and *Necator americanus* (human hookworm) are the two worms that have been investigated as therapeutic agents for diseases as wide ranging as autism, multiple sclerosis, asthma, allergic rhinitis, celiac disease, Crohns disease, and ulcerative colitis. FDA-regulated clinical trials are ongoing for the treatment of Crohns disease with *Trichuris suis* ova (TSO). While these large trials will establish if treatment is efficacious, a small pilot mechanistic trial on ulcerative colitis patients is also ongoing to investigate the mechanism of action for TSO in the intestinal tract [10]. Recently, it has become clear that the gut microbiota plays an important role in establishing a balanced immune response and is often dysregulated in autoimmune diseases [22]. In addition to the mechanisms already discussed above, helminth exposure may also restore the gut microbiota to a healthy state [23], which will enhance the immune regulatory network of the mammalian host [24].
